# Psychopathic traits are related to diminished guilt aversion and reduced trustworthiness during social decision-making

**DOI:** 10.1038/s41598-019-43727-0

**Published:** 2019-05-13

**Authors:** Xu Gong, Inti A. Brazil, Luke J. Chang, Alan G. Sanfey

**Affiliations:** 1 Center for Brain Disorder and Cognitive Science, Shenzhen University, Shenzhen, China; 2 Shenzhen Key Laboratory of Affective and Social Cognitive Science, Shenzhen University, Shenzhen, China; 3 College of Psychology and Sociology, Shenzhen University, Shenzhen, China; 4 Donders Institute for Brain, Cognition, and Behaviour, Radboud University, Nijmegen, The Netherlands; 5 Forensic Psychiatric Centre Pompestichting, Nijmegen, The Netherlands; 6 Department of Psychological & Brain Sciences, Dartmouth College, Hanover, NH USA; 7 Behavioural Science Institute, Radboud University, Nijmegen, The Netherlands

**Keywords:** Computational models, Human behaviour

## Abstract

Individuals with high levels of psychopathic tendencies tend to show a lack of guilt, a lack of empathic concern, and a disregard for the impact of their decisions on others. However, how guilt influences social decision-making for those with high psychopathic traits is still unknown. Here, we investigated how psychopathic traits relate to the capacity to acquire knowledge about social expectations, and to what extent guilt aversion affects subsequent decision-making. 63 participants completed self-report measures of psychopathy, and then played a modified Trust Game in the role of the Trustee. Results showed that participants’ self-reported beliefs about their partner’s expectations were largely predictive of the amount of money they returned to the partner. These decisions were negatively correlated with the PPI-I scores. Furthermore, participants’ degree of guilt aversion were negatively correlated with PPI total scores. Our findings suggest that individuals with higher psychopathic traits are indeed capable of understanding the expectations of others, but do not seem to directly utilise this knowledge in their social decision-making, and experience less anticipated guilt about this. The present study provides empirical evidence of intact social knowledge coupled with decreased reciprocity and diminished guilt aversion as levels of psychopathic traits increase.

## Introduction

Psychopathy is a personality construct characterized by impaired social-emotional processing combined with a tendency to display disruptive and antisocial behaviors^1^. The interpersonal-affective disturbances that lie at the core of this construct encompass a lack of empathy, guilt, and remorse^2–4^, and are considered to be unique to psychopathy. Psychopathy, with its distorted moral reasoning, transgressive actions, as well as deficits in social functioning, has been widely acknowledged as a risky social threat that leads to insecurity and unrest, arousing fear across society^5^. Moreover, disturbed behaviors associated with psychopathy are more broadly distributed in non-clinical populations with high psychopathic traits^6–9^. These traits can be measured using self-report questionnaires, such as the Psychopathic Personality Inventory (PPI)^10^ and the Self-Report Psychopathy Scale-Short form (SRP-SF)^11^. It is of vital importance for scientific investigations to better understand the psychological processes and mechanisms underlying psychopathic behaviors in the general population, which can also then provide novel insight into the violent mind.

Psychopathy has repeatedly been linked to poor social decision-making, partly hypothesized as due to a diminished capability for making appropriate social inferences and for following social norms and rules^12–14^. For example, the prisoner’s dilemma game (PDG), in which two players simultaneously and independently choose to either cooperate or defect based on reciprocal altruism or reciprocal exchange of favors, is typically used to measure cooperation. The optimal individual decision for a player in terms of pay-off is to always choose ‘defect’ as compared to ‘cooperate’, though this is considered a less prosocial choice. Previous studies have found that offenders with psychopathy showed deficits in reasoning about social rules during social exchange while being asked to predict the opponent’s behavior before making their own decision in an iterated PDG^15^. In addition, higher levels of psychopathy have been associated with more selfish patterns of cooperation with others in both offenders and non-offenders^9,16^.

Altruistic punishment occurs when individuals punish social norm violators even when this behavior is costly for the punisher^17^ Research has revealed that individuals with higher psychopathic traits give greater punishment to other players who treat them unfairly as compared to individuals with lower psychopathic traits, and that these punishment decisions were positively correlated to self-reported emotional gratification scores rather than sympathy scores. A mediational analysis further revealed that the predictive effect of psychopathy on emotional gratification was mediated by altruistic punishment, which suggested that the source of the punishment by the individuals with high psychopathic traits may lie in their own emotional gratification or satisfaction^18^. Furthermore, individuals with high psychopathic traits reduced their use of social advice during decision-making^19^. Together, these findings highlight different aspects of impaired social decision-making that have been associated with increasing levels of psychopathy. Understanding more about these impairments is essential, especially because they often lead to negative consequences for others^13,19^.

For optimal decision-making, choices made during social exchange require accurate predictions of the expectations of one’s social partner. Yet, i) it is unclear to what extent individuals with elevated levels of psychopathy possess the capacity to make the social inferences required to undergo a mutually satisfactory exchange; ii) or, whether they do have the necessary knowledge of the expectations of their interaction partner, but are simply insensitive to this social knowledge. Hypothetical moral dilemmas scenarios have previously been used to assess willingness to harm others and reason about transgressions, in comparison to non-psychopathic samples^20,21^. For a particular moral dilemma scenario, on the one hand, people may feel the action is wrong; on the other hand, a cost-benefit analysis is calculated in which action can lead to a better outcome. For example, people usually report the reluctance to push a human down a footbridge to stop a trolley from running over five persons. The former is a direct ‘personal’ harm, which is associated with strong negative emotional responses, eliciting moral disapproval. The latter is an indirect ‘impersonal’ harm, which sacrifice one for preserving the welfare of a large number of other people, reflecting greater concern for a rational ‘utilitarian’ decision than the emotionally aversive means^22,23^. The utilitarian moral response pattern among individuals with high psychopathic traits may reflect a reduced influence of affective processes on decision-making that leads to lessened interest in the expectation and welfare of others, even when they are capable of acquiring social knowledge^24–26^. However, a recent study using these social dilemmas found no differences in utilitarian moral judgment between psychopaths and a control group^27^. Hence, hypothetical moral dilemma scenarios are a limited way to answer the controversial question of whether, and to what degree, psychopaths have restricted understanding of moral knowledge and expectations of others that are used to guide judgments and decisions.

**Figure 1:**
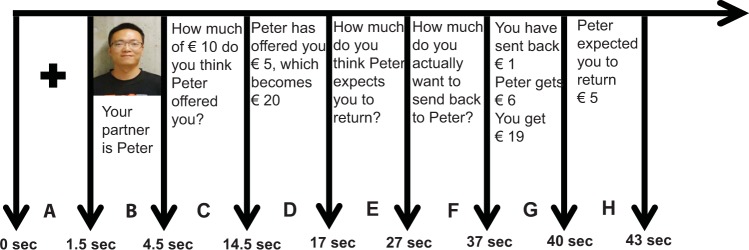
The timeline of a single round of the modified Trust Game. (**A**) Each round begins with a fixation cross in the middle of the screen. (**B**) Facial pictures of Player 1 on that round. (**C**) Participants indicate their belief about Player 1’s investment. (**D**) The actual offer from Player 1. (**E**) Participants indicated their belief about what Player 1 had expected them to return. (**F**) Participants indicated the amount of money they actually wanted to return. (**G**) Payoff for both players revealed. (**H**) The real expectation of Player 1 on that round revealed.

Here, we employed a model of guilt aversion as assessed by a Trust Game paradigm (Fig. 1) in order to quantify our participants’ knowledge of the expectations of others, their guilt aversion, and their social decision-making. The guilt aversion model has shown that the anticipation of guilt may be one of the potential mechanisms that motivates cooperative behavior in many social settings^28^. This study found that beliefs about the expectations of others motivated reciprocal behavior, and that counterfactual guilt (defined as the amount of guilt individuals would have felt had they returned less money than they believed their partner expected) also promoted reciprocal behavior. According to this model, one’s aversion to the possibility of experiencing guilt in the future prompts reciprocal decisions in the present in order to minimize this anticipation of guilt, and this can be a powerful motivator in the decision-making process. Since guiltlessness is a key feature of psychopathy, it might well be expected that high levels of psychopathic traits relate to diminished guilt aversion, and that this may play a key role in the pattern of social decisions seen in psychopathy. The general goal of the present study was to systematically explore the relationship between psychopathic traits in a non-offender sample, the generation of social inferences, and guilt aversion during social decision-making. Firstly, we investigated whether the norm violations and socially inappropriate behavior typically seen in relation to increasing levels of psychopathy were associated with a reduced understanding of social norms and expectations, or rather whether understanding is intact but that these norms and expectations are taken less into account during decision-making. Given the absence of behavioral performance differences between individuals high and low in psychopathic traits in moral and social decision-making tasks^27,29,30^, we hypothesized that non-incarcerated individuals scoring relatively high on psychopathy would be unimpaired in understanding others’expectations, but nevertheless would choose to ignore this social information when making reciprocal decisions^19^. Secondly, we examined the relationship between guilt aversion and reciprocity. Considering that individuals with high levels of psychopathic traits are more likely to engage in non-normative social behavior and express less guilt and concern about others’ emotions or welfare, we hypothesized that increasing levels of psychopathic traits should correspond to less guilt aversion and thus lessened reciprocal behavior during social decision-making. Importantly, there is evidence that the nature of the relationships between psychopathy and measures of cognitive functioning is highly dependent on the measurement of psychopathy used^31,32^. Therefore, we employed two instruments, the PPI and the SRP-SF, which are widely used for measuring psychopathy-related traits in community samples (Table 1). The PPI was specifically designed to assess psychopathic personality traits among the non-clinical population, and discerns eight specific constituent traits(s) and provides a continuum measurement of each trait. In addition, the SRP-SF, derived from the PCL-R (Psychopathy Checklist-Revised)^33^, mainly used for estimating the maladaptive behaviors among the clinical psychopathic population, was also used as a supplemental measure. We hypothesized that the PPI scores may more sensitive and predictive when assessing psychopathy traits among nonforensic (e.g., community and students) samples as compared to the SRP-SF^34^.

**Table 1:** Mean PPI and SRP (scale and subscale) scores (n = 63) with corresponding standard deviations (SD).

Variable	Mean (*SD*)		
	All (63)	Female (50)	Male (13)
Age	22.14 (3.85)	21.68 (0.44)	23.92 (1.57)
Total PPI scores^a^	335.25 (58.33)	332.26 (5.21)	376.23 (9.03)
PPI-I (or Fearless Dominance)^a^	126.38 (20.29)	124.38 (2.76)	134.08 (6.18)
PPI-II (or Antisocial impulsivity)^a^	167.70 (23.94)	162.28 (3.25)*	188.54 (3.98)*
Stress immunity^a^	26.22 (6.02)	25.72 (0.84)	28.15 (1.72)
Social potency^a^	59.03 (11.14)	58.54 (1.62)	60.92 (2.81)
Fearlessness^a^	41.13 (8.49)	40.12 (1.07)	45.00 (3.00)
Cold-heartedness^a^	43.41 (8.22)	41.84 (1.06)	49.46 (2.29)
Blame externalisation^a^	33.63 (8.50)	32.44 (1.18)	38.23 (2.15)
Carefree non-planfulness^a^	39.75 (6.07)	38.64 (0.84)	44.00 (1.26)
Machiavellian egocentricity^a^	58.56 (12.27)	56.60 (1.69)	66.08 (3.02)
Impulsive nonconformity^a^	35.76 (7.13)	34.60 (1.01)	40.23 (1.41)
Total SRP scores^b^	47.08 (13.97)	43.52 (1.55)	60.77 (4.49)
SRP-F1^b^	23.46 (8.83)	21.18 (0.94)*	32.23 (2.99)*
SRP-F2^b^	23.62 (6.30)	22.34 (0.77)	28.54 (1.99)
SRP-Interpersonal^b^	12.30 (5.64)	11.24 (0.63)*	16.38 (2.11)*
SRP-Affective^b^	11.16 (3.84)	9.94 (0.41)	15.85 (0.98)
SRP-Lifestyle^b^	13.97 (4.25)	13.10 (0.55)	17.31 (1.12)
SPR-Antisocial^b^	9.65 (3.06)	9.24 (0.37)	11.23 (1.14)

*Significant differences between Male and Female sample.

^a^PPI^10^, ^b^SRP-SF^58^.

## Results

### General results

**Figure 2:**
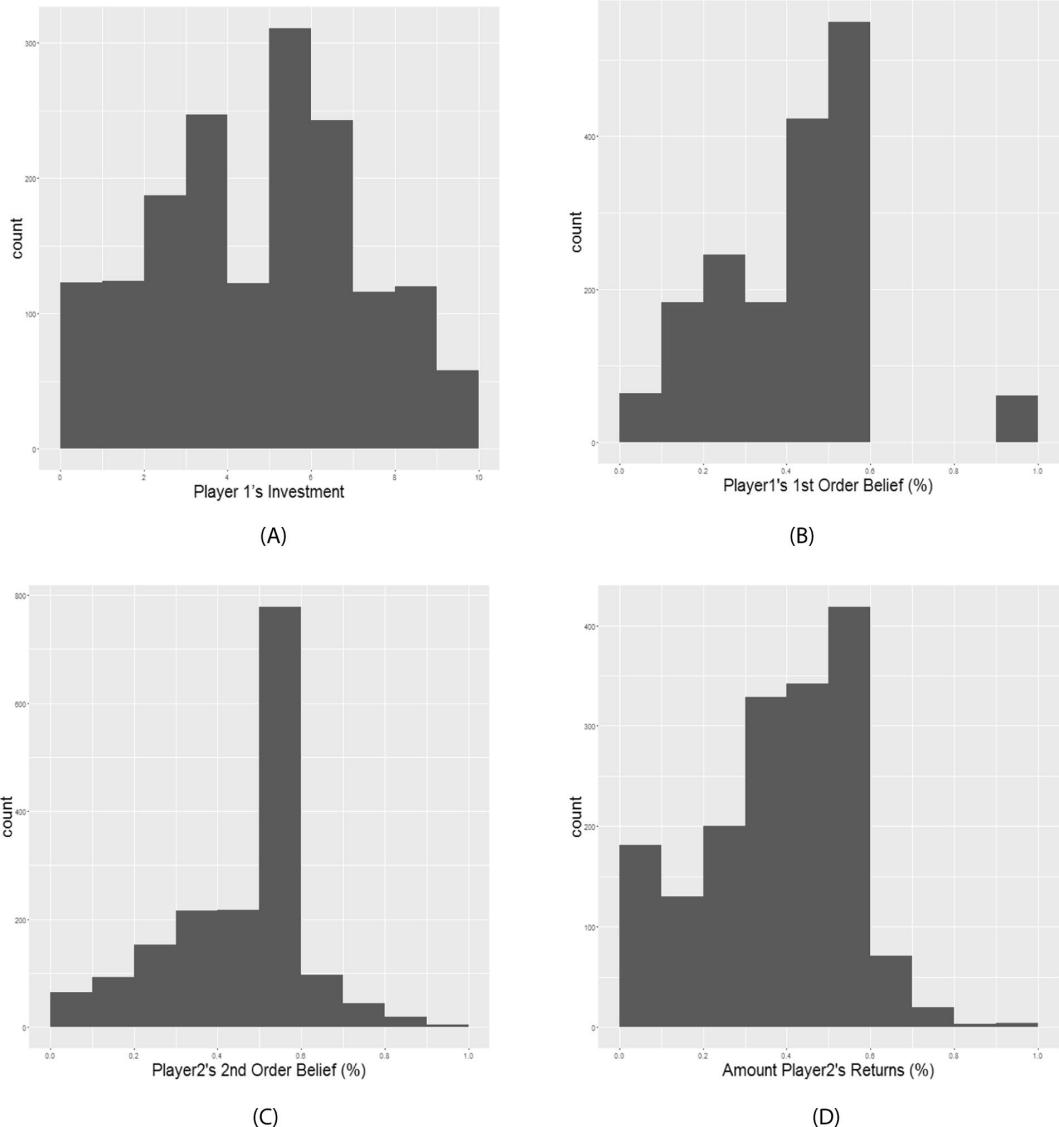
Descriptive statistics of the Behavioural Results. (**A**) Histogram of Player 1’s Investment across all trials (*M* = 48.3%, *SD* = 28.7%). (**B**) Histogram of the percentage of their investment (multiplied by 4) that Player 1 expects Player 2 to return (Player 1’s 1^st^ Order Belief) (*M* = 39%, *SD* = 17%). (**C**) Histogram of the percentage of the transferred amount (multiplied by 4) that Player 2 believes Player 1 expects them to return (Player 2’s 2^nd^ Order Belief) (*M* = 44%, *SD* = 17%). (**D**) The percentage of the transferred amount that Player 2 decides to return (*M* = 36%, *SD* = 18%).

**Figure 3:**
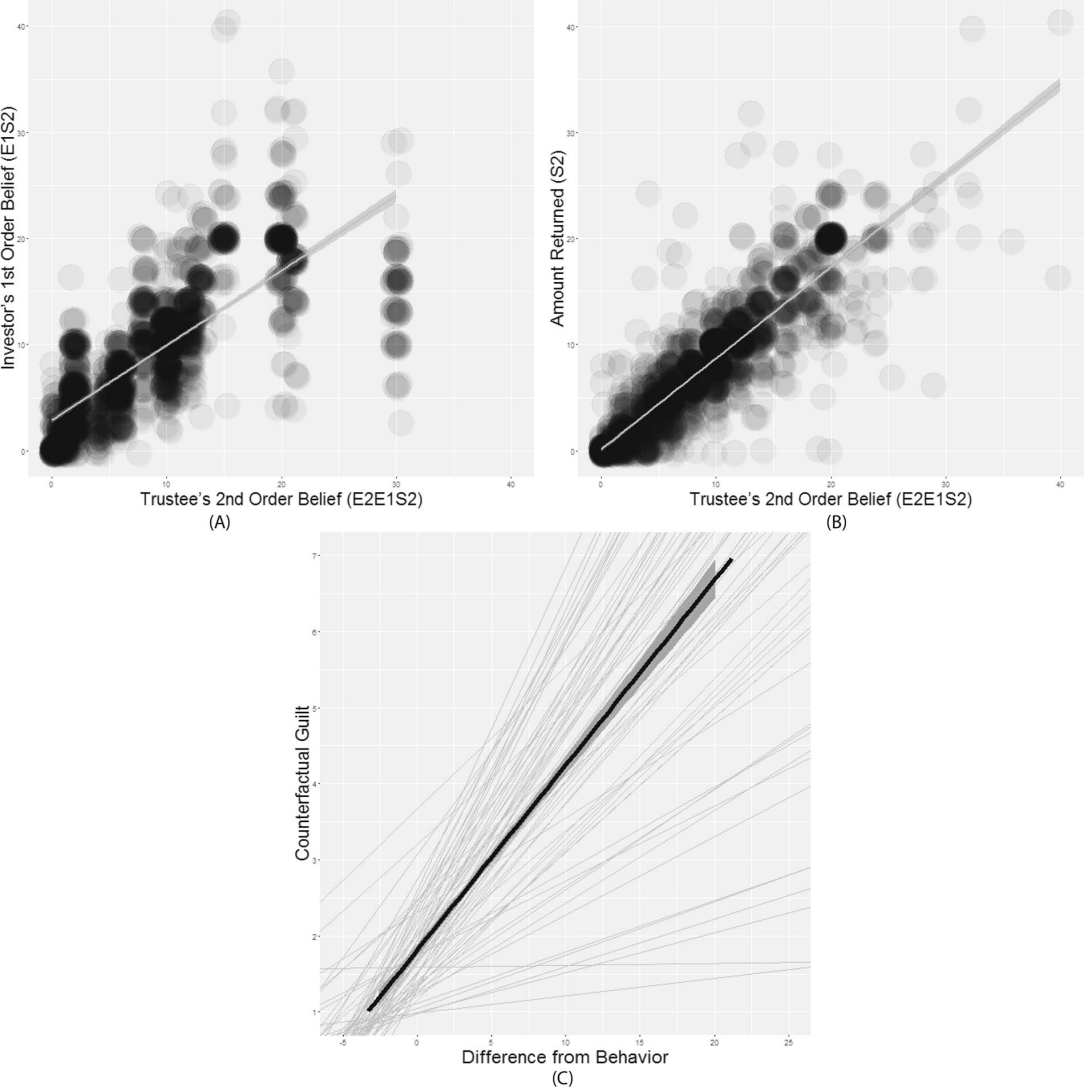
Relationships between performance measures. (**A**) Player 1’s 1^st^ Order Belief (*E*_1_*S*_2_) by Player 2’s 2^nd^ Order Belief (*E*_2_*E*_1_*S*_2_). (**B**) The amount returned by the Trustee (Player 2) (*S*_2_) by their 2^nd^ Order Belief. (**C**) Player 2’s self-reported counterfactual guilt (the amount of guilt they would have felt had they returned less money) by the difference between their hypothetical choices from their actual behaviors. The dotted lines represent the participants’ best linear unbiased predictions (BLUPs).

On average across all trials, Player 1 invested 48.3% (*SD* = 28.7%) of their endowment (Fig. 2A), and their expectation (Player 1’s 1^st^ order belief) was that Player 2 would return 39% of the total investment (*SD* = 17%) (Fig. 2B). Player 2 believed that Player 1 expected them to return on average 44% (*SD* = 17%) of the total investment (Fig. 2C). The average percentage of the total investment that Player 2 actually decided to return was 36% (*SD* = 18%) (Fig. 2D). Mixed-effects regression results indicated that participants in the role of Player 2 could accurately predict Player 1’s expectations (1^st^ Order belief) (*B* = 0.70, *SE* = 0.02, *t* = 37.80, *p* < 0.001) (Fig. 3A). In addition, Player 2’s then appeared to use their predicted Player 1’s expectation (2^nd^ Order Belief) to guide their decisions, in that they typically returned the amount of money that they believed Player 1 expected them to return (*B* = 0.87, *SE* = 0.02, *t* = 42.02, *p* < 0.001) (Fig. 3B). Furthermore, participants reported that they would experience more counterfactual guilt if they had returned less money than they actually did (*B* = 0.23, *SE* = 0.02, *t* = 15.5, *p* < 0.001) (Fig. 3C). Taken together, these results replicate previous findings, supporting the guilt aversion model^28^.

### Correlation between psychopathy measures and behavioral performance

To examine the relationship between decision-making and variations in psychopathic traits, we computed the correlation coefficients, and corresponding bootstrapped confidence intervals, between participants’ PPI and SRP scores (total, as well as the two main factors) and participants’ behavioral responses in the Trust Game (Table 2). Significant negative correlations were found between participants’ self-reported counterfactual guilt and their PPI total scores (*r* = −0.33), PPI Factor 1 scores (*r* = −0.25), and PPI Factor 2 scores (*r* = −0.29). This indicates that individuals who scored higher on psychopathic traits reported feeling less counterfactual guilt if they would have had returned less money to Player 1. Furthermore, individuals with higher scores on the PPI-I scales actually returned less money to Player 1 (PPI-I: *r* = −0.26). Moreover, the partial correlation analyses yielded non-significant results (*see* Supplement Table S1). Therefore, only PPI scores were used in subsequent analyses.

**Table 2:** Correlation coefficients and bootstrapped confidence intervals between PPI/SRP scores and behavioral responses (*r* values, 95% confidence intervals).

	PPI			SRP		
	Total	Factor I	Factor 2	Total	Factor 1	Factor 2
CFGuilt	−**0**.33** [−0.53, −0.10]	−**0**.25* [−0.46, −0.02]	−**0**.29* [−0.51, −0.04]	−0.09 [−0.41, 0.24]	−0.15 [−0.45, 0.18]	−0.18 [−0.44, 0.13]
PredInvest (%)	−0.08 [−0.33, 0.17]	−0.13 [−0.34, 0.09]	−0.01 [−0.27, 0.24]	0.11 [−0.15, 0.37]	0.03 [−0.20, 0.28]	−0.03 [−0.25, 0.21]
PredReturn (%)	0.11 [−0.09, 0.31]	0.02 [−0.18, 0.23]	0.11 [−0.11, 0.32]	0.09 [−0.13, 0.31]	0.14 [−0.09, 0.37]	0.16 [−0.09, 0.41]
P2Retrun (%)	−0.22 [−0.43, 0.01]	−**0**.26** [−0.42, −0.12]	−0.13 [−0.41, 0.16]	0.00 [−0.26, 0.28]	0.01 [−0.24, 0.29]	0.02 [−0.20, 0.28]

*p < 0.05 level (2-tailed), **p < 0.01 level (2-tailed). CFGuilt: participants’ self-reported amount of counterfactual guilt they would have felt had they returned less money. PredInvest (%): the percentage of Player 1’s investment (multiplied by 4) that Player 2 believes Player 1 expects them to return. PredRetrun (%): the percentage of Player 1’s investment (multiplied by 4) that Player 2 believes the Player 1 expects them to return. P2Return (%): the percentage of Player 1’s investment (multiplied by 4) that Player 2 actually decides to return.

### Correlations between computational parameters, behavior and PPI scores

We tested whether the guilt aversion model could predict participants’ decisions. Across all participants, the mean of the parameter θ_12_ was 0.43 (*SD* = 0.17, range = [0.13,0.91]) and the mean of $\phi$ was 19.59 (*SD* = 19.93, range = [4.47,104.11]). Next, bootstrapped Pearson correlations were computed between the two free parameters and the behavioral responses, as well as PPI (total and factors) scores (see Table 3). A significant positive relationship was detected between the parameter θ_12_ and the amount of counterfactual guilt (*r* = 0.38) as well as between θ_12_ and the percentage of Total Amount actual returned (*r* = 0.72). This suggests that individuals who are more likely to report counterfactual guilt actually return more in the task, and also had higher guilt sensitivity parameter estimates. Furthermore, the parameter θ_12_ was significantly negatively correlated with the PPI total score (*r* = −0.28), indicating that higher levels of psychopathic traits corresponded to reduced guilt sensitivity. For the parameter $\phi$, we only found a significant positive relationship with the counterfactual guilt (*r* = 0.27).

**Table 3:** Correlation coefficients (r) and 95% bootstrapped confidence intervals (between brackets) for the relationship between the computational parameters, behavioral measures and PPI scales.

	Behavior			PPI	
	CFGuilt	P2Return (%)	Total	Factor 1	Factor2
θ_12_	**0**.38** [0.15, 0.58]	**0**.72** [0.51, 0.86]	−**0**.28* [−0.50, −0.03]	−0.22 [−0.45, 0.03]	−0.23 [−0.48, 0.04]
*φ*	**0**.27* [0.07, 0.45]	0.06 [−0.34, 0.46]	−0.22 [−0.50, 0.11]	−0.14 [−0.40, 0.14]	−0.19 [−0.47, 0.12]

Note: *p < 0.05 level (2-tailed), **p < 0.01 level (2-tailed). CFGuil: participants’ self-reported the amount of counterfactual guilt they would have felt had they returned less money. P2Return (%): the percentage of Player 1’s investment (multiplied by 4) that Player 2 actually decided to return.

## Discussion

In this study, the role of guilt aversion during social decision-making in relation to psychopathic tendencies was studied in a non-clinical sample. The results indicated that elevated levels of psychopathic traits are not associated with a reduced understanding of social norms and expectations, but rather that these norms and expectations are taken less into account during social decision-making. Moreover, guilt aversion played a less prominent role in the decision-making process as the level of psychopathic tendencies increased.

The findings demonstrated a negative association between guilt aversion and PPI total scores. Additionally, participants’ self-reported counterfactual guilt showed a negative relationship with the PPI total and factor scores. Finally, reciprocal behavior, indexed by the percentage of the total investment that participants actually returned, correlated negatively with participants’ PPI-I scores. These experimental findings support the hypothesis that individuals scoring higher on psychopathic personality traits do indeed possess accurate knowledge about others’ social expectations. However, the diminished reciprocal behavior shown by these individuals indicate that they are less likely to use this knowledge when making monetary decisions in a social context^19^, and that a reduced aversion to anticipated feelings of guilt plays a role in driving this tendency to disregard the expectations of others.

The relationship between the capacity for social inferencing, decision-making, and psychopathy has typically been studied in the context of moral reasoning. Many studies on this topic have employed hypothetical moral dilemmas to study how psychopathy might be related to judgments about the appropriateness of a given choice that affects others^24,35^. These studies provided initial indications that individuals with a high level of psychopathy may show more ‘utilitarian’ moral decisions. One explanation for these results could be that these individuals are not capable of properly incorporating social-affective information to guide their decisions^36^. However, other studies have found that having a high level of psychopathy is related to an intact capacity to discern right from wrong^27,37^, as well as an unaffected capacity to make social inferences such as those concerning the intentionality of others’ actions when making considerations about fairness^38^. Our results are in agreement with the latter body of findings, as they indicate that the capacity to generate beliefs about others’ expectations does not diminish as the level of psychopathic traits increases.

We also found that a high level of psychopathy is associated with less aversion to anticipated feelings of guilt when generating monetary choices based on social beliefs, in general agreement with previous findings. It has been proposed that it is the affective component of decision-making which is impaired in psychopathy rather than the cognitive component^39^.

Blair^12^ suggested the processing of social-affective information (e.g., sad or fearful facial expressions) signaling when one’s own choices have detrimental outcomes for others is impaired in psychopathy, and that this impairment interferes with social and moral decision-making^20,40^. Indeed, some studies have found diminished physiological reactions to negative emotional stimuli (e.g., fearful and angry faces) among psychopathic offenders during passive observation as compared to nonpsychopathic controls^41,42^. Other recent investigations have attempted to pinpoint how deficient processing of social-affective information translates to impaired social decision-making as a function of psychopathy level. For example, Marsh and Cardinale^36^ reported that psychopathy was associated with a reduced ability to recognize behaviors that cause fear in others, and moral transgressions based on these behaviors were more likely to be rated as being acceptable. Another study found that increased utilitarian tendencies as the presence of psychopathic traits increases were partly driven by a reduced aversion to engaging in actions that are harmful to others^43^. Finally, Seara-Cardoso and colleagues^44^ discovered that the modulation of anticipation of guilt by the insula interacted with the interpersonal features of psychopathy. Our finding that guilt aversion decreases as psychopathy scores increase is in agreement with the notion that impairments in the affective domain interfere with other cognitive operations subserving social decision-making. Furthermore, the formal approach employed in our study provides significant advances as it allows us to disentangle and quantify separate cognitive processes within a well-defined and validated mechanism describing the nature of their interactions during social decision-making.

One critical note is that we should remain cautious in extrapolating our results, obtained in a healthy sample, to incarcerated populations with severe levels of psychopathology. Nonetheless, as psychopathy has become more accepted as a constellation of personality traits, dimensional analyses with larger sample sizes would provide novel and valuable angles for further understanding the in-depth nature of psychopathy. Another point is that gender distribution was not optimal in our sample, though there is evidence showing that empathic processing and moral reasoning in fact do not differ between genders as a function of psychopathy scores in community samples^45,46^. This adds support to the validity of our results, but due to the novelty of our approach, it remains important to replicate our results in larger, gender-balanced, samples in the future.

A final issue is that our auxiliary measure of psychopathy (the SRP-SF) failed to yield any significant correlations, and thus led to different results as compared to the PPI. This is in accordance with studies showing that the specific psychopathy instrument used has a large effect on the nature of both the significance and direction of the correlations. Across previous studies, the relationship between psychopathic traits and executive functions were divergent when assessing psychopathic traits using PCL-R and Multidimensional Personality Questionnaire (MPQ), that estimated the factors of psychopathy within a broad inventory of normal personality functioning^31^. Moreover, individuals with high psychopathic traits have also shown inconsistent results in executive and social functions^27^. One potential reason might be that different measures may be qualitatively and quantitatively non-comparable though assessing a similar factor of psychopathy. In our case, this lack of effect is not entirely unsurprising given that we oversampled the upper and lower extremes of the distribution of psychopathy scores based on the PPI total scores. While this approach helps to maximize the inclusion of relatively extreme PPI scores in the experimental sample, it does not lead to a similar effect for the distribution of the SRP-SF scores. Note also that the PPI and the SRP show strong differences in how they conceptualize and measure psychopathy, especially at the factor level^47^. Thus, it is likely that our oversampling procedure to maximize the tails of the PPI total score distribution had a suboptimal effect on the distribution of SRP (factor) scores. The distribution of the SRP-SF total score in our sample (see Supplementary Fig. S3) confirmed this explanation that most of our participants dropped to the lower SRP total score distribution. Moreover, the SRP-SF, derived from the PCL-R, has been examined, though in a limited way, in terms of the factor structure in community samples. However, findings have been inconsistent^34,48^. As compared to PPI, it is mainly used for estimating maladaptive behaviors among clinical psychopathy samples. Ongoing development of the SRP scale has involved adding a greater number of antisocial behavior items as compared to the PPI^34^. Our correlation results support the suggestion that SRP scores (both the total score and the two main factor scores) are correlated with the PPI-II rather than PPI-I. The PPI-I is the factor that represents the interpersonal (e.g., charm, grandiosity, and deceitfulness), and affective (e.g. lack of remorse, empathy, and emotional depth) traits of psychopathy, which reflect low guilt and deficient emotion processing^49^. In contrast, the PPI-II is the factor that captures the impulsive and chronic antisocial tendencies associated with psychopathy, and is related to the inhibition control deficit. It is therefore reasonable that the PPI-I is more relevant to the current interpersonal decision-making context as compared to the PPI-II. Our results also support this idea in that a significant positive predictive effect was detected between the PPI-I score and the subsequent reciprocal decision-making (see Results), but a similar effect was not found with the PPI-II.

In conclusion, the present study showed that individuals scoring high on psychopathic traits are able to accurately infer the social expectations of others when making monetary choices in a social context, but that they are still more prone to demonstrate selfish, less-reciprocal, decision-making. In our study, two separate measures of participants’ guilt sensitivity were used; self-reported counterfactual guilt and a computational parameter capturing guilt sensitivity for each participant. Across both measures, we found that the higher people scored on PPI total scores, the lower their guilt aversion. These results support the hypothesis that an attenuated sensitivity to social emotions (in this case anticipated guilt) among individuals scoring high on psychopathy is related to indifference to others’ welfare and profit, and leads them to ignore social expectations. That is, we propose that individuals with high levels of psychopathic traits have the ability to understand social expectations, but down-weight this information when making social decisions^50–52^.

## Methods

### Participants

Sixty-six participants were recruited via two different rounds at Radboud University, the Netherlands (*see* Supplement). They received either monetary compensation (16 Euros) or course credit (3 points) for completing the task. Additionally, participants had the opportunity to receive a monetary bonus dependent on task performance (maximum of 10 Euros). The study ethical approval has been granted by the ethical committee of the Faculty of Social Sciences at the Radboud University (ECG2012-1304-025). All participants provided written informed consent in accordance Declaration of Helsinki and the guidelines of the local ethics committee.

Three participants were excluded from the statistical analyses; two did not finish the experiment in time, and the third because they did not complete the PPI and SRP questionnaires. Therefore, analyses were performed on 63 participants (50 females), with age ranging from 18 to 40 (*M* = 22.28, *SD* = 4.03). For an additional check of gender-dependent distribution of scores, a two-sample t-test was performed between the female and male group for all the PPI/SRP total and subscales (see Table 1).

### Measures of psychopathy-related traits

The Dutch translation of the PPI was used as the primary measure of psychopathy^10,53^. The PPI is a comprehensive self-report instrument that estimates psychopathic personality characteristics in non-clinical samples. The PPI consists of 187 items, measuring traits on 8 subscales that represent different general personality traits. Each item is evaluated on a 4-point Likert-scale, ranging from 1 (false) to 4 (true). Higher PPI total scores correspond to higher degrees of psychopathy. Factor analyses have found that the scales of the PPI load on super-ordinate factors, known as the PPI-I (Fearless Dominance) and the PPI-II (Antisocial impulsivity)^49^. Three subscales, namely Social Potency, Fearlessness, and Stress Immunity, are clustered into the PPI-I Factor; while four other subscales - Machiavellian Egocentricity, Blame Externalisation, Carefree Non-planfulness, and Impulsive Nonconformity - comprise the PPI-II Factor. A third factor consists of the subscale Coldheartedness. The total PPI score is usually regarded as a global index of the psychopathy, with strong internal consistency (Cronbach’s alpha 0.89)^54^. PPI factor scores were calculated by first normalizing to raw scores (z-transformation) based on the total experimental sample size and summing the relevant scales to create the PPI-I and PPI-II factors, respectively.

The Dutch version of Hare’s Self-Report Psychopathy scale was used as an additional measure to assess psychopathy-related traits^55^. The SRP-SF is directly derived from the Psychopathy Checklist-Revised (PCL-R)^33^, a semi-structured clinical interview that is considered to be the golden standard for assessing psychopathy. The SRP-SF differs from the PPI in that it measures maladaptive behaviors and traits instead of general personality traits. The SRP-SF consists of 29 items that measure 4 facets of psychopathy; Interpersonal (Facet 1) and affective disturbances (Facet 2), antisociality (Facet 3), and deviant lifestyle (Facet 4). Facets 1 and 2 can be clustered into a higher-order factor measuring interpersonal-affective impairments (SRP-F1) and facets 3 and 4 can be combined into an antisocial-lifestyle factor (SRP-F2). The two-factor model has been the most prominent in the literature. The SRP-SF has good psychometric properties (Cronbach’s alpha 0.84)^55^.

### Experimental task

A modified version of the Trust Game was used^28^. The game is played by two players, with Player 1 being the ‘investor’ and Player 2 the ‘trustee’. Player 1 is first endowed with a sum of money by the experimenter (€10 in our experiment). Player 1 then decides the amount of money he/she wants to invest with Player 2, in 1 Euro increments ranging from zero to the entire endowment. Once an amount has been selected by Player 1, this is multiplied by 4, and this new amount (i.e., the total investment) is transferred to Player 2. Importantly, both players know in advance about the multiplier. Following the transfer, Player 2 has the opportunity to return some of this transferred money back to the Investor, but is not obliged to. The amount that Player 2 decides to return to Player 1 was our primary behavioral index in this game. In each round, Player 2 was also asked to estimate how much he/she believed that Player 1 would invest and how much he/she believed Player 1 expected in return (Fig. 1). All participants played in the role of Player 2.

After participants had completed the experiment, they were sequentially shown the outcomes of the 30 rounds with all Player 1’s and were asked to rate their counterfactual guilt on a 7-point Likert scale (scaled 1–7, with 1 indicating not feeling any guilt, 4 indicating moderate guilt, and 7 indicating feeling extremely guilty), on which they could indicate the amount of guilt they believed they would have experienced if they had returned a different amount of money. This ‘counterfactual’ amount was randomly selected from all choices lower than the amount they had returned and one choice higher than the amount they had returned (choices increased or decreased by 10% increments). Finally, all participants were paid a bonus based on their decisions in one randomly selected round.

### Guilt aversion model

We fit the data with an adapted guilt aversion model^28^ to estimate individuals’ guilt sensitivity. This model proposes that the utility of Player 2 in the Trust Game could be divided into two parts: financial utility (*M*_2_) and the anticipated experience of guilt (or guilt utility). A rational Player 2 is interested in balancing both parts through maximising monetary income and minimising anticipated guilt. In our task, anticipated guilt can be considered as the non-negative difference between the amount of money Player 1 expects Player 2 to return (*E*_1_*S*_2_) and the amount of money Player 2 actually returns (*S*_2_). Since Player 2 does not know the amount Player 1 expects them to return (1^st^ order belief), the model uses Player 2’s belief of Player 1’s expectation (2^nd^ order belief: *E*_2_*E*_1_*S*_2_) as a substitute input.

The mathematical derivation of these parameters is described in equation (1). Player 2’s total utility in trial *i* is *U*_2*i*_, which can be defined as

1 $${U}_{2i}=(1-{\theta }_{12}){M}_{2i}-{\theta }_{12}{({E}_{2}{E}_{1}{S}_{2}-{S}_{2})}^{+}$$

According to the above model, the relative weight placed on the financial payoff (*M*_2_) and the anticipated guilt (*E*_2_*E*_1_*S*_2_ − *S*_2_)^+^ in the utility function is modulated by a guilt sensitivity parameter (*θ*_12_), which can vary for each Player 1 that Player 2 meets. This guilt sensitivity term is scaled by a free parameter, constrained in the range of ($0 < {\theta }_{12} < 1$). The θ_12_ (guilt sensitivity) parameter quantifies how sensitive participants are to the experience of guilt if they let their partner down. For example, if θ_12_ = 0, they are completely self-interested, demonstrating insensitivity to guilt; while if θ_12_ = 1, they are very sensitive to anticipated guilt. We theorize that balancing the weights of both financial income (self-interest) and Player 2’s (2^nd^ order belief) plays a decisive role in guiding decisions in the Trust Game.

### Choice rule

We extended the above model to allow for stochasticity (i.e., randomness) by applying a probabilistic choice function. A free parameter $\phi$ was used to capture the stochasticity in the action selection. The probability *P*_*i*_ of making a decision *i* was computed by placing the utility values for each decision into the following softmax function.

2 $${P}_{i}=\frac{{{\rm{e}}}^{{\phi }_{i}{U}_{2i}}}{{\sum }_{k=0}^{K}\,{{\rm{e}}}^{{\phi }_{i}{U}_{2i}}}$$

in which *P*_*i*_ denotes the probability that in trial *i* Player 2 chooses *k* (with *k* standing for the maximum amount of money in that trial they could return) to return to Player 1. The free parameter ${\phi }_{i}$ is used to capture the sensitivities of choice *i* to different utilities. If ${\phi }_{i}$ = 0, choices are random, while if ${\phi }_{i}$ = Infinity, the utility is maximised. In the guilt aversion model (Equation 1), the total utility is comprised of both the participants’ income (*M*_2_) and the weighted anticipation of guilt (*E*_2_*E*_1_*S*_2_ − *S*_2_)^+^. The model of guilt sensitivity included a stochastic of the choice sensitivity parameter $\phi$, in which both parameters were estimated using maximum likelihood estimators (for details *see* Supplement).

### General analyses

Frequency distributions of the following variables across all trials over all participants are shown in Fig. 2: (A) Player 1’s Investment; (B) The percentage of Player 1’s investment (multiplied by 4) that participants expected Player 2 to return (1^st^ Order Belief: *E*_1_*S*_2_); (C) The percentage of Player 1’s investment (multiplied by 4) that Player 2 believed Player 1 had expected them to return (Player 2’s 2^nd^ Order Belief: *E*_2_*E*_1_*S*_2_); (D) The percentage of Player 1’s investment (multiplied by 4) that Player 2 actually decided to return (*S*_2_); Furthermore, mixed model regressions (*see* Supplement) were implemented between (A) Player 1’s 1^st^ Order belief and Player 2’s 2^nd^ Order belief. Significant effects here would indicate that participants were capable of accurately inferring other players’ expectations; (B) Player 2’s 2^nd^ Order belief and the amount of money they returned. Significant effects here would illustrate that participants’ behavioral patterns are inconsistent with a guilt aversion model to a certain extent. In addition, for each participant, mixed effect regression was used to predict the amount of counterfactual guilt reported if they returned a different amount of money. According to the guilt aversion model, the deviation between the participants’ actual repayment and their belief about Player 1’s expectation indexed anticipated guilt. Thus, each participant’s best linear unbiased predictions (BLUPs) demonstrate their sensitivity to guilt. The group BLUP was computed based on individual scores. Larger slopes indicated higher guilt sensitivity.

### Analyses of relationships with psychopathy traits

**Figure 4:**
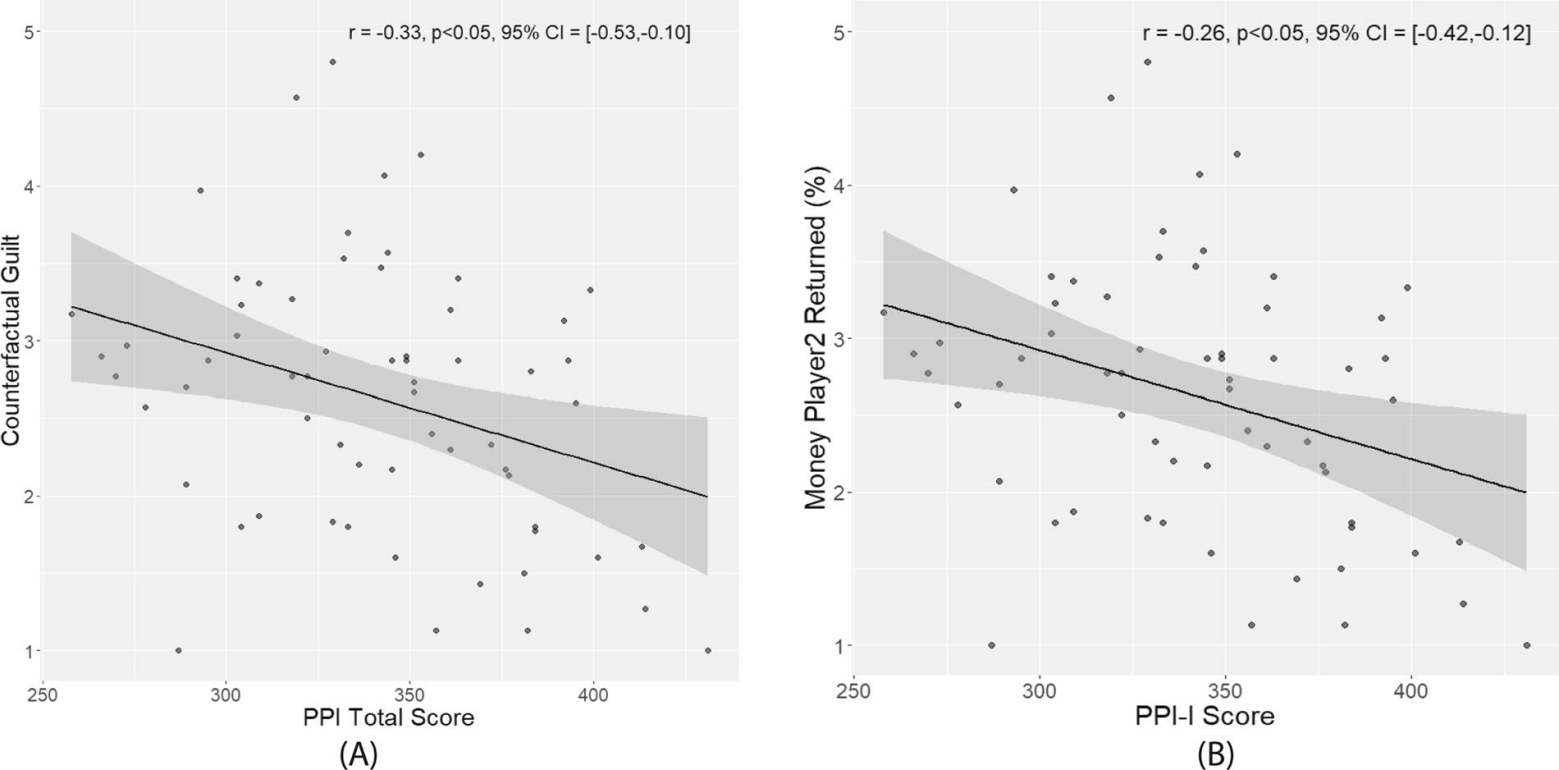
Pearson correlation between scores on PPI/SRP and behavioural performance. (**A**) Scatterplot for Pearson correlations between total PPI score and participants’ reported counterfactual guilt. (**B**) Scatter plot for Pearson correlations between PPI Factor 1 score and the percentage of the total investment that Player 2 returned.

To understand the relationships between psychopathic traits as well as the participants’ utilitarian behaviors, Pearson correlations were computed between the global indexes of PPI/SRP scores (the total as well as the two main factor scores) and participants’ behavioral responses (counterfactual guilt and amount of money actually returned) (Table 2 and Fig. 4). Because the SRP measures correlated factors known to have suppression effects^56^, we also ran partial correlation analyses to control for the shared variance between the factors. The significance of the Pearson correlations was tested with a bootstrapping procedure to determine the 95% confidence interval (CI). The same procedure was used to obtain Pearson correlations for the relationship between the two estimated free parameters θ_12_ and φ, the participants’ behavioral responses, and the total and factor scores of the PPI and the SRP, respectively^57^.

## Supplementary information

Supplement document

## Data Availability

The datasets generated during and/or analysed during the current study are available from the corresponding author on reasonable request.
