# Supplementary material for: Psychopathic traits are related to diminished guilt aversion and reduced trustworthiness during social decision-making
Source: Sci Rep. 2019 May 13;9:7307. doi: 10.1038/s41598-019-43727-0 (PMC6514208; doi:10.1038/s41598-019-43727-0)
Supplement: Supplementary file 1 — Supplement document [file 41598_2019_43727_MOESM1_ESM.docx]

### ***Supplement-*Psychopathic traits are related to diminished guilt aversion and reduced trustworthiness during social decision-making**

Xu Gong*^1,2,3,4^, Inti A. Brazil^4,5^, Luke J. Chang^6^, Alan G. Sanfey^4,7^

^1^Center for Brain Disease and Cognitive Science, Shenzhen University, Shenzhen, China

^2^Shenzhen Key Laboratory of Affective and Social Cognitive Science, Shenzhen University, Shenzhen, China

^3^College of Psychology and Sociology, Shenzhen University, Shenzhen, China

^4^Donders Institute for Brain, Cognition, and Behaviour, Radboud University, the Netherlands

^5^Forensic Psychiatric Centre Pompestichting, Nijmegen, the Netherlands

^6^Department of Psychological & Brain Sciences, [Dartmouth College](https://scholar.google.nl/citations?view_op=view_org&hl=en&org=18229439179148490080), Hanover, NH, USA

^7^Behavioural Science Institute, Radboud University, the Netherland

***Correspondence author notation**:

Xu Gong, PhD

**Mail address**: Donders Institute for Brain, Cognition, and Behaviour, Radboud University, Kapittelweg 29, 6525 EN, Nijmegen, The Netherlands.

**Email address**: x.gong@donders.ru.nl

### ***Participant recruitment***

Recruitment occurred in two rounds. During the first round, 141 participants (including a non-student sample, 105 females, mean age=22.22, SE=6.11) were screened using the PPI and SRP. Afterwards, PPI total score quartiles were used to split the participants into four groups. Twenty-three participants (16 females, mean age=22.91, SE=1.05) from the two extreme (i.e., lowest and highest) quartile groups were selected to join the experiment. Of these, 11 participants had scored high on psychopathic traits (HPT) and 12 had scored low on psychopathic traits (LPT). Comparing PPI scores, the HPT group (*M*=409.36, *SD=*48.3) had significantly higher total scores than the LPT group (*M*=307.17, *SD*=24.3, *t* (21)=1.01, *p<.001)*. During the second round, 43 participants took part in the experiment, irrespective of their PPI scores. Some of the participants filled out the PPI and SRP questionnaires online at home prior to the experimental session, while others filled out the questionnaires during the experimental session.

***Data analyses and statistical packages***

Participant-specific best-fitting parameters $\theta_{12}$ and $\varphi$ were estimated using nonlinear parameter optimisation, which were then implemented in the MATLAB fmincon function ^1^ for trial-wise maximum log likelihood estimation. The estimation was run separately for each individual. Thus, we obtained the best fitting parameters $\theta_{12}$ and $\varphi$ for each participant.

Statistical analyses were performed using the R statistical package ^2^ and MATLAB R2014a (MathWorks, MA). The “lme4” package was used for the mixed effect regression with repeated measures ^3^, in which participants were treated as the random effect. To examine the robustness of the results, the *p*-values in the mixed effect model were computed in three ways; with (i) standard approximation, (ii) Satterthwaite approximation using the “lmerTest” package ^4^ and (iii) Kenward-Roger approximation using the “pbkrtest” package ^5^. Consistent results were found. The “psych” package was used for the bootstrapping correlation. The significance of correlation was tested with a non-parametric bootstrapping procedure (10000 samples) to determine the confidence interval (CI). If a correlation is significant its CI should not include the value of 0, which means that both the upper and lower bound of a CI should be either larger or smaller than 0.00. We used the “ggplot2” package for visualisation of the results ^6^. We maximised the log-likelihood of the data under each model on a trial-wise basis.

***Additional task details***

For Player 1, 30 transfer amounts, along with their expectations of the returned amount, were taken from one of our previous studies ^7^. Each Player 1 had an identification number assigned to their actual facial photographs. Participants were informed that the set of Player 1s they would encounter were other participants whose data had been previously collected. Each round began by showing participants a picture of Player 1. In each round, they would play with one of the 30 different Player 1s (Figure 1). Firstly, they were asked to estimate how much money Player 1 had invested with them. Then, participants saw the actual transfer amount, and were asked how much money they thought Player 1 would expect them to return. Next, they decided on the amount of money (from zero to the maximum of the transferred money) they wanted to return. Notably, participants had the opportunity not to return any money. A summary of the outcome containing the final payment for that round for both players was presented on the screen at the end of each trial, and the amount expected by Player 1 for that round was also revealed to the participant.

Participants made their decisions using a keyboard. Participants pressed Key 1 to select the amount of money they would like to return and pressed Key 2 to confirm their choice. The amount returned changed in 10% increments on each key press. These increments were randomly selected to either increase from €0 or decrease from the maximum amount of money for that round (which varied depending on offer amount by the partner).

Stimulus presentation and data acquisition were conducted using E-Prime software and VisuaStim goggles (Resonance Technologies Inc., IL, USA). After having made sure that participants fully understood the instructions, participants were given the opportunity to practice several trials (not included in the statistical analyses).

**Table S1**. Partial correlations for the SRP scales (*r* values, 95% confidence intervals)

|  |  | SRP |  |
| --- | --- | --- | --- |
|  | Total | Factor 1 | Factor 2 |
| CFGuilt | 0.11  [-0.09, 0.3] | 0.07  [-0.13, 0.26] | -0.02  [-0.18, 0.22] |
| PredInvest (%) | 0.15  [-0.05, 0.34] | 0.06  [-0.14, 0.25] | -0.03  [-0.25, 0.17] |
| PredReturn (%) | 0.03  [-0.17, 0.23] | 0.08  [-0.12, 0.28] | 0.07  [-0.13, 0.26] |
| P2Retrun (%) | 0.1  [-0.1, 0.3] | 0.14  [-0.06, 0.33] | 0.13  [-0.07, 0.32] |

CFGuilt: participants’ self-reported amount of counterfactual guilt they would have felt had they returned less money. PredInvest (%): the percentage of Player 1’s investment (multiplied by 4) that Player 2 believes Player 1 expects them to return. PredRetrun (%): the percentage of Player 1’s investment (multiplied by 4) that Player 2 believes the Player 1 expects them to return. P2Return (%): the percentage of Player 1’s investment (multiplied by 4) that Player 2 actually decides to return.

**Table S2.** Correlations between the PPI and SRP scales (*r* values, 95% confidence intervals)

|  | PPI-Total | PPI-I | PPI-II | SRP-Total | SRP-F1 | SRP-F2 |
| --- | --- | --- | --- | --- | --- | --- |
| PPI-Total | 1 |  |  |  |  |  |
|  |  |  |  |  |  |  |
| PPI-I | 0.78^***^ | 1 |  |  |  |  |
|  | [0.65,0.87] |  |  |  |  |  |
| PPI-II | 0.83^***^ | 0.34^**^ | 1 |  |  |  |
|  | [0.75,0.89] | [0.12,0.55] |  |  |  |  |
| SRP-Total | 0.58^***^ | 0.22 | 0.67^***^ | 1 |  |  |
|  | [0.41,0.74] | [-0.04, 0.49] | [0.56,0.78] |  |  |  |
| SRP-F1 | 0.56^***^ | 0.20 | 0.66^***^ | 0.95^***^ | 1 |  |
|  | [0.37,0.73] | [-0.08, 0.47] | [0.53,0.77] | [0.90,0.97] |  |  |
| SRP-F2 | 0.49^***^ | 0.21 | 0.57^***^ | 0.89^***^ | 0.69^***^ | 1 |
|  | [0.32,0.67] | [-0.03, 0.49] | [0.42,0.70] | [0.80,0.89] | [0.48,0.83] |  |

**p* < .01 level (2-tailed), ** *p* < .001(2-tailed).


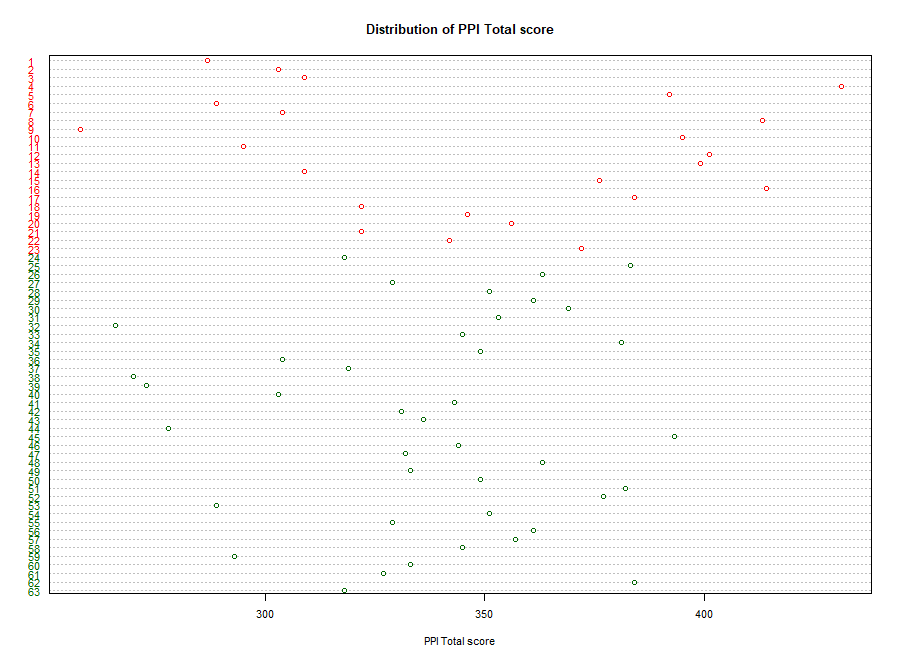


**Figure S1**. The Distribution of the PPI Total Score from the two rounds of participants. The scatter plot of the red dots shows the distribution of the first round of 23 participants (on the upper part of the figure), and the green dots indicates the distribution of the second round of 43 participants (on the lower part of the figure)


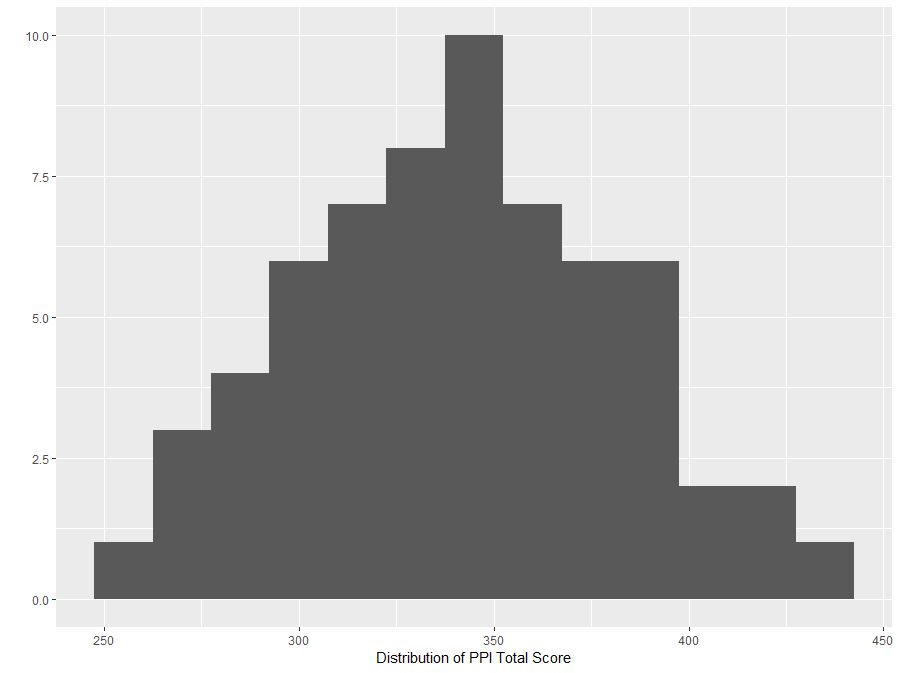


**Figure S2**. the Distribution of the PPI Total Score of our sample (N=63)


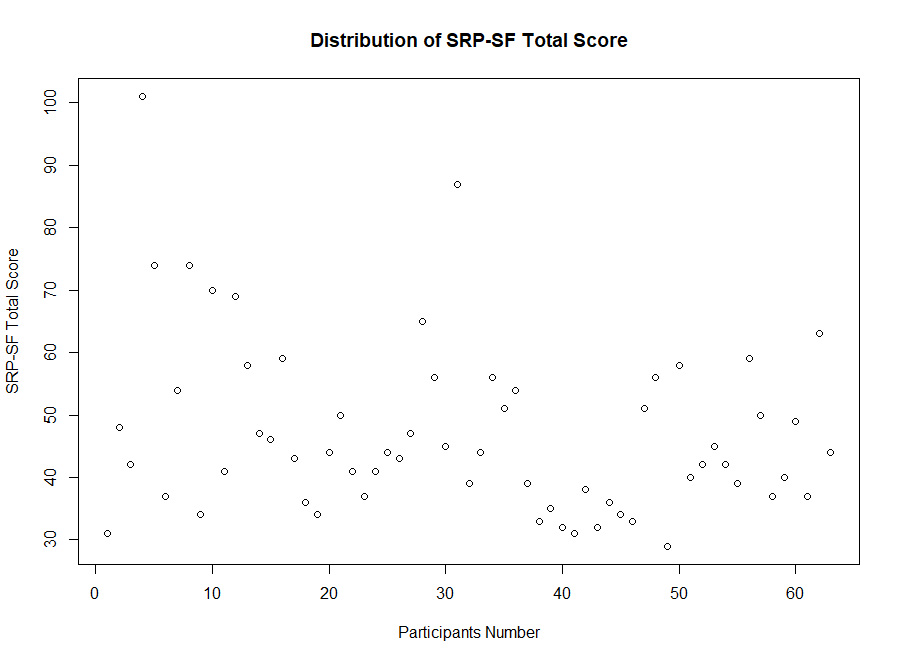


**Figure S3.** The Distribution of SPR-SF Total Score

**References**

1 Nelder, J. A. & Mead, R. A Simplex-Method for Function Minimization. *Comput J* **7**, 308-313 (1965).

2 R Core Team. (Vienna, Austria, 2015).

3 Bates, D., Mächler, M., Bolker, B. & Walker, S. Fitting Linear Mixed-Effects Models Using lme4. *Journal of Statistical Software* **67**, 1-48 (2015).

4 Kuznetsova, A., Bruun Brockhoff, P. & Haubo Bojesen Christensen, R. (2015).

5 Halekoh, U. & Højsgaard, S. A Kenward-Roger Approximation and Parametric Bootstrap Methods for Tests in Linear Mixed Models - The R Package pbkrtest. *Journal of Statistical Software* **59**, 1-30 (2014).

6 Wickham, H. *ggplot2: Elegant Graphics for Data Analysis*. (Springer-Verlag New York, 2009).

7 Chang, L. J., Smith, A., Dufwenberg, M. & Sanfey, A. G. Triangulating the neural, psychological, and economic bases of guilt aversion. *Neuron* **70**, 560-572, doi:10.1016/j.neuron.2011.02.056 (2011).
